# Assessment of Vancomycin Penetration into Cerebrospinal Fluid in Patients with Ventriculitis Using a Physiologically Based Pharmacokinetic Approach

**DOI:** 10.1007/s11095-026-04023-5

**Published:** 2026-02-04

**Authors:** Larissa Martins Alves Guimarães, Letícia Oliveira de Vasconcellos Nacif, Jhohann Richard de Lima Benzi, Mayra Torres de Oliveira Santos, Carolina Yamada, Felipe Francisco Tuon, João Paulo Telles, Fernanda de Lima Moreira

**Affiliations:** 1 Laboratory of Pharmacometrics (LabFarma), Faculty of Pharmacy, Federal University of Rio de Janeiro, Rio de Janeiro, 21941-902 Brazil; 2 Department of Pharmacy, School of Pharmaceutical Sciences, University of São Paulo, São Paulo, Brazil; 3 Escola de Medicina, Pontifícia Universidade Católica do Paraná, Curitiba, Paraná Brazil; 4 Department of Infectious Diseases, AC Camargo Cancer Center, São Paulo, 01509 010 Brazil

**Keywords:** brain, central nervous system, PBPK, vancomycin

## Abstract

**Introduction:**

Vancomycin is an antimicrobial agent for treating central nervous system (CNS) infections caused by Gram-positive bacteria. Due to practical and ethical reasons, it is difficult to evaluate vancomycin exposure in cerebrospinal fluid (CSF) and its relationship with therapeutic outcomes. Therefore, alternative methodologies are required. We developed a physiologically based pharmacokinetic (PBPK) model to characterize vancomycin exposure in plasma and CSF in patients with ventriculitis enrolled in a therapeutic drug monitoring program.

**Methodology:**

PBPK modeling and simulation were conducted using PK-Sim® version 11.3. A PBPK model was constructed to simulate vancomycin exposure in plasma and CSF. Physicochemical parameters of vancomycin were incorporated into a large molecule model, and tissue distribution was described using the Rodgers-Rowland model.

**Results:**

The final PBPK model incorporated vancomycin’s low brain permeability by adjusting the CSF-to-plasma partition coefficient to 0.17. Model validation was performed using data from 33 patients with ventriculitis under external ventricular drainage. The dosing regimen consisted of a 30 mg/kg loading dose followed by a continuous intravenous infusion of 60 mg/kg/day. Mean simulated vancomycin concentrations in plasma and CSF were 32 mg/L and 7.2 mg/L, respectively. The predicted CSF/plasma concentration ratio was 0.22, which closely matched the observed ratio of 0.17.

**Conclusion:**

Vancomycin penetration into the CNS is low and variable, highlighting the importance of therapeutic drug monitoring and individualized therapy in patients with ventriculitis. In the future, this model may facilitate the selection of optimal dosing regimens by simulating alternative dosing strategies and establishing PK/PD relationships for CNS infections.

**Supplementary Information:**

The online version contains supplementary material available at 10.1007/s11095-026-04023-5.

## Introduction

After the early 1960 s, the clinical use of vancomycin was found to be associated with significant toxicities, mainly renal injury. However, with improvements in the manufacturing process of the molecule and the implementation of therapeutic drug monitoring, its toxicity has decreased. Vancomycin remains the gold standard for the treatment of Methicillin-resistant *Staphylococcus aureus* (MRSA) according to the latest guidelines [1]. Additionally, the emergence and spread of multidrug-resistant bacteria have underscored the importance of vancomycin as a valuable therapeutic option [2].

Optimal vancomycin dosing remains a subject of debate in the medical literature. Factors such as age, renal function, and disease severity influence the choice of dose and dosing frequency. A major advancement in vancomycin monitoring has been the adoption of the area under the concentration–time curve (AUC) as the primary parameter for evaluating treatment efficacy [3]. This shift reflects progress in scientific understanding and the pursuit of more precise and effective strategies to treat resistant bacterial infections, highlighting the need for individualized vancomycin therapy to optimize clinical outcomes and minimize toxicity risks.

In infections such as meningitis and ventriculitis, which affect the central nervous system (CNS), higher serum vancomycin levels may be required to ensure therapeutic efficacy, given its limited penetration into this site [3]. Ventriculitis is an infection of the brain's ventricular system, characterized by the presence of ventricular pleocytosis (increased leukocytes), bacteria, and reduced glucose levels in the patient's cerebrospinal fluid [4]. The external ventricular drainage (EVD) is a closed drainage system designed to drain cerebrospinal fluid (CSF) in cases of hydrocephalus and ventricular hemorrhage. However, there is a risk of infection associated with the use of this device [4] that increases with its prolonged use.

It is essential to distinguish between the clinical syndromes of meningitis and ventriculitis, as they differ markedly in terms of blood–brain barrier (BBB) and blood–CSF barrier integrity, which directly impacts vancomycin permeability into the CNS. In meningitis, these barriers are typically disrupted due to intense inflammatory processes, facilitating increased vancomycin penetration into the CNS. In contrast, ventriculitis may develop in patients without significant barrier disruption, particularly in cases of acute non-traumatic intracerebral hemorrhage requiring EVD for decompression, where the structural integrity of the BBB and CSF barriers is often preserved, thereby limiting CNS penetration of vancomycin. Lastly, in patients with ventriculitis following EVD placement after traumatic brain injury, data remain scarce regarding the extent of barrier disruption, and it is unclear whether vancomycin permeability in this subgroup more closely resembles that observed in meningitis or in patients with intact barriers. As a result, achieving therapeutic concentrations in the CSF may be more difficult in ventriculitis, rendering the treatment in this context particularly challenging [5, 6].

The search for strategies to optimize vancomycin therapy to improve the management of CNS infections remains a crucial area of research [7]. Current approaches are largely based on general pharmacokinetic data, with a lack of (i) evidence demonstrating clinical impact and (ii) clearly defined pharmacodynamic (PD) targets [8]. Furthermore, CSF drug concentrations are often interpreted in isolation as indicative of therapeutic adequacy, without sufficient consideration of the complex clinical context associated with CNS infections [9]. However, CSF concentrations do not necessarily correlate with tissue drug levels, and this misconception may lead to unjustified modifications in therapeutic regimens, potentially increasing the risk of drug-related toxicities. For instance, antibiotic concentrations may differ across CNS compartments: the lateral ventricles, where an EVD is placed, typically exhibit one of the highest levels, whereas lumbar CSF generally has the lowest [10].

In this context, physiologically based pharmacokinetic (PBPK) modeling has emerged as a powerful tool to simulate drug disposition in the human body, offering a deeper understanding of pharmacokinetic processes. By representing the body as a system of interconnected compartments, PBPK models simulate drug absorption, distribution, metabolism, and excretion while accounting for patient-specific physiological characteristics. By integrating physiological and biochemical data with mathematical modeling, PBPK models enable accurate predictions of drug concentrations in both plasma and tissues, supporting dose optimization, formulation development, and drug interaction assessments. However, the complexity of biological systems and the scarcity of experimental data still pose challenges for the construction and validation of these models [11]. The ability to incorporate the CNS compartment, including CSF, into PBPK models may enhance the understanding of vancomycin penetration into this site and contribute to advancing knowledge of the pharmacokinetic-pharmacodynamic relationship in the treatment of meningitis and ventriculitis with vancomycin. Considering this, this study aimed to develop a physiologically based pharmacokinetic (PBPK) model to characterize vancomycin exposure in plasma and lateral ventricular CSF in patients with ventriculitis enrolled in a therapeutic drug monitoring (TDM) study.

## Methods

### Clinical Protocol, Ethical Aspects and Sample Collection

The TDM study was conducted after approval of the Ethics Committee of the Pontifical Catholic University of Paraná (CAAE number 75526017.0.0000.0020). Participants were included in the research after signing the Informed Consent Form. Patients included in the study followed the criteria: admission to the intensive care unit and prescription of vancomycin for presumed or documented ventriculitis. Patients with vancomycin allergy and those under 18 years of age were excluded from the study. The baseline characteristics of the study population (*n* = 33) are demonstrated in Table I.

**Table I:** Baseline Characteristics of the Study Population (n = 33). Data Are Presented As Median (Q1–Q3) for Continuous Variables and N (%) for Categorical Variables

Variable	Value
Demographic and systemic variables	
Male sex, *n* (%)	19 (56%)
Female sex, *n* (%)	15 (44%)
Age, years	54.5 (39.8–61.8)
CSF Lactate, mmol/L	5.4 (3.8–7.2)
CSF characteristics	
CSF glucose, mg/dL	62.0 (37.5–104.5)
CSF leukocytes, cells/mm^3^	145.0 (71.0–540.0)
CSF neutrophils, %	67.0 (53.0–86.5)
CSF protein, mg/dL	154.0 (68.0–243.0)
Comorbidities, *n* (%)	
HIV infection	0 (0%)
Diabetes without chronic complications	5 (15%)
Diabetes with chronic complications	1 (3%)
Any diabetes (with or without complications)	6 (18%)
Chronic renal failure on dialysis	2 (6%)
History of myocardial infarction	1 (3%)
Heart failure (NYHA class III)	0 (0%)
Peripheral vascular disease	0 (0%)
Cerebrovascular disease	1 (3%)
Hemiplegia	2 (6%)
Dementia	1 (3%)
Chronic obstructive pulmonary disease	1 (3%)
Hypertension	12 (36%)
Any neoplasm	7 (21%)
Lymphoma	0 (0%)
Leukemia	0 (0%)
Metastatic solid tumor	0 (0%)
Rheumatologic disease	0 (0%)
Peptic ulcer disease	0 (0%)
Mild liver disease	0 (0%)
Cirrhosis with hepatic encephalopathy	0 (0%)
Outcome, *n* (%)	
Death	15 (45%)
Discharged alive	19 (57%)

*CSF* Cerebrospinal fluid

The strategy for blood and ventricular CSF sampling is outlined as follows. After at least 24–48 h from the start of continuous infusion, blood and ventricular cerebrospinal fluid samples were obtained. Ventricular CSF was collected directly through the external ventricular drain to determine the vancomycin concentration in the CSF and inflammatory parameters. Blood was collected from a venipuncture in an EDTA tube and immediately centrifuged and serum was obtained. Serum and CSF samples were analyzed immediately after collection. The vancomycin in plasma and CSF samples was measured using a commercial kit for vancomycin quantification using particle-enhanced turbidimetric inhibition immunoassay using a Siemens Dimension Xpand Plus HM clinical analyzer (Siemens, Munich, Germany). The curve in each biological fluid was built with vancomycin concentration at 0, 2, 5, 10, 20, and 40 mg/L in blank plasma or CSF, evaluated in duplicate. The lower limit of quantification was 2 mg/L and the limit of detection was 0.8 mg/L. The intra- and inter-day coefficients of variation values were ≤ 20% for 2 mg/L and ≤ 15% for the other concentration points.

Thirty-three patients were included in the study, all patients received 30 mg/kg of vancomycin as a loading dose followed by 60 mg/kg continuous dose using an infusion pump (e.g., 24 h) for 10–14 days. Renal function was continuously monitored, glomerular filtration rate was calculated using the Chronic Kidney Disease Epidemiology Collaboration (CKD-EPI) formula, and clinical and laboratory data were analyzed. The demographic and biochemical parameters of this population study were published by Tuon *et al*. [12].

### Physiologically-based Pharmacokinetic Model Development

A full-body PBPK model for vancomycin was developed using PK-Sim® software version 11.3 (Open Systems Pharmacology) using the large molecule module. The physicochemical and absorption, distribution, metabolism and excretion (ADME) input data (Table II) for vancomycin were adapted from Rezende *et al*. [13]. The vancomycin PBPK model development, refinement and validation followed the best practices recommended by the Organisation for Economic Co-operation and Development (OECD) guideline [14] and are fully described in Rezende *et al*. [13].

**Table II:** Input Parameters for Human Physiologically Based Pharmacokinetic (PBPK) Model for Vancomycin Adapted from Rezende et al. [13]

Parameter (unity)	Value
Molecular weight (g/mol)	1449.265
log P	−3.10
pKa	2.18 (acid), 7.75 (base)
Blood to plasma ratio	0.75
Fraction unbound to plasma protein	0.67
Water solubility (mg/mL)	100
Partition coefficient	Rodgers and Rowland
Cellular permeability	PK-Sim standard
(cm/min)^a^	0.01
Brain permeability plasma to interstitial space (cm/min)	0.17 ± 0.14
Hepatic clearance (mL/min/kg)	0.42
Glomerular Filtration Rate	1.0*

^*^indicating elimination by glomerular filtration pathway

Virtual patients were created in PK-Sim, incorporating data on the number of individuals, female proportion, age, height, weight and renal function (measured by creatinine values) according to the present TDM study and Albanese *et al*. [15].

#### Vancomycin PBPK Brain Model

**Fig. 1:**
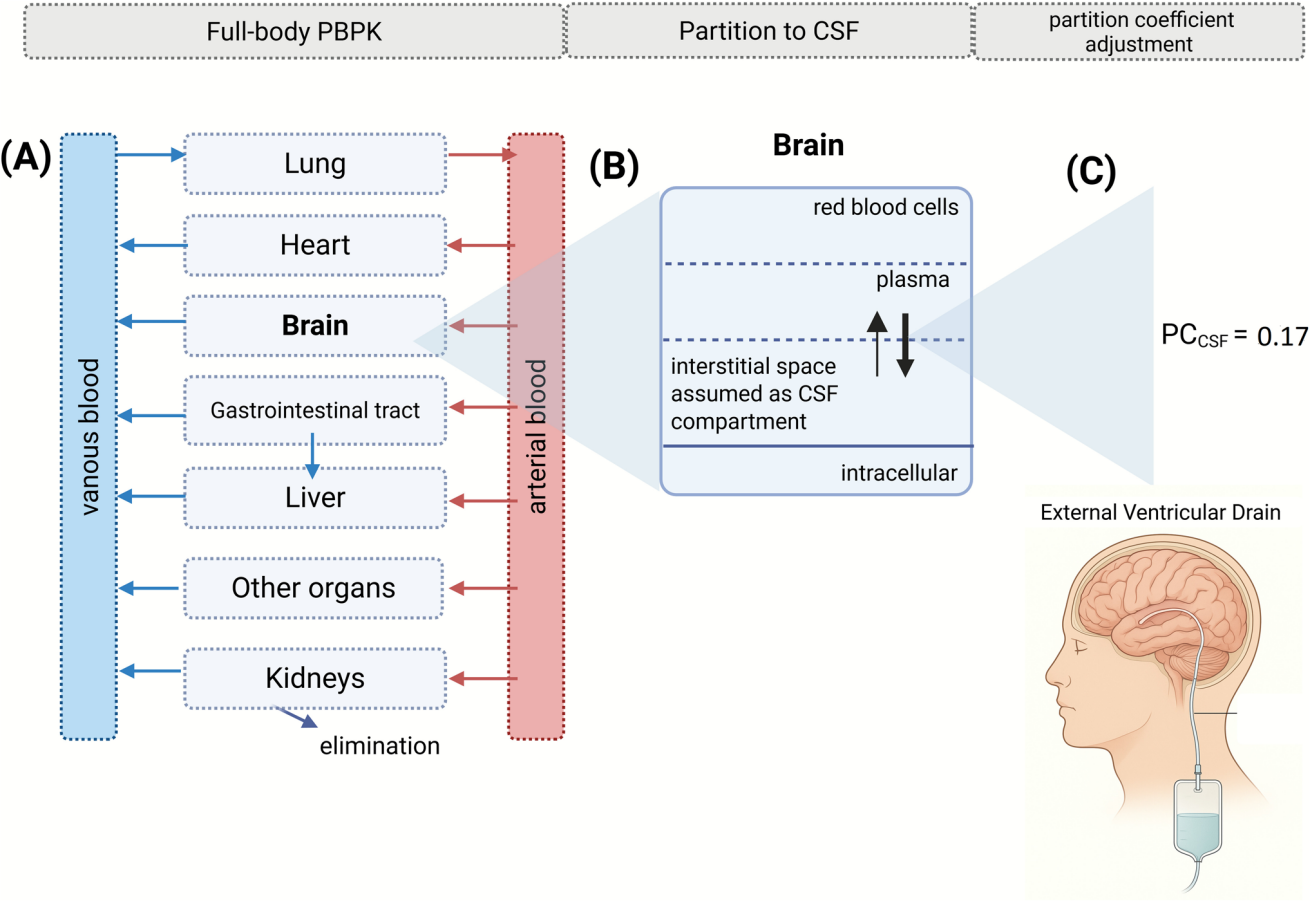
Schematic overview of Physiologically-based Pharmacokinetic (PBPK) modeling workflow. (**A**) Development of a vancomycin full-body PBPK; (**B**) Development of a brain model with interstitial space representing the cerebrospinal fluid (CSF) compartment, (**C**) refinement of the brain model with plasma-to-brain interstitial partition coefficient (PC_CSF_) value of 0.17 (dimensionless).

A schematic overview of vancomycin PBPK modeling workflow is demonstrated in Fig. 1. The brain was represented using four default subcompartments in PK-Sim: blood, plasma, interstitial fluid (ISF) (representing interventricular CSF), and intracellular space. These subcompartments were connected to the full PBPK model via the arterial and venous blood compartments.

To identify the specific brain parameters that influence vancomycin distribution to CSF subcompartment. The sensitivity was calculated as following Eq. 1 [16]:

1 $$S=\frac{\Delta PK}{\Delta p}\times \frac{p}{PK}$$

where *S* is the sensitivity, *PK* is the initial value of the pharmacokinetic parameter, Δ*PK* is the change of the pharmacokinetic parameter from the initial value, *p* is the initial value of the examined parameter, and Δ*p* is the change of the examined parameter from the initial value, respectively. A sensitivity of + 1.0 indicates that a + 10% change of an examined input parameter causes a + 10% change in the predicted pharmacokinetic parameter. A sensitivity analysis, including 38 specific brain input data, fraction unbound to plasma protein and fraction of water in plasma, was generated and only parameters different from zero were considered relevant.

After this, the plasma-to-brain interstitial partition coefficient (PC_CSF_) was the only parameter considered relevant in vancomycin PBPK brain model refinement. The PC_CSF_ is calculated in the simulator as the following Eq. 2 [16]:

2 $${PC}_{CSF}=\left({Fw}_{CSF}+{Albumin}_{CSF:plasma}\times \left(\frac{1}{fup}-{Fw}_{plasma}\right)\right)\times fup$$

where, Albumin_CSF:plasma_ is the albumin CSF to plasma ratio, fup is the fraction unbound in plasma, Fw_CSF_ is the fraction of water in CSF, Fw_plasma_ is the fraction of water in plasma. The ${PC}_{CSF}$ value calculated considering a perfusion-limited model was 0.77.

To adjust the vancomycin permeability to the ISF, the parameter identification tool provided by PK-Sim®, using the Levenberg–Marquardt algorithm, was applied to find the optimal value within a specific range to minimize the residuals between the simulation output and the actual observed values from the TDM study used during the model refinement step.

Model validation was considered successful when the observed PK profile was within the 5th and 95th percentiles of predicted data, and the predicted steady-state CSF concentration to steady-state plasma concentration ratios were within a 0.5- to twofold range compared to the observed data [14]. Mean relative deviation (MRD) demonstrated in Eq. 3 was used for quantitative evaluation of all plasma and CSF concentration predictions. MRD values ≤ 2 were interpreted as signs of adequate model performance.

3 $$\mathrm{MRD}={10}^{\mathrm{x}};\text{ x}=\sqrt{{\sum }_{\mathrm{i}=1}^{\mathrm{m}}{\frac{\left({\mathrm{log}}_{10\mathrm{cpredicted},\mathrm{i}}- {\mathrm{log}}_{10\mathrm{cobserved},\mathrm{i}}\right)}{\mathrm{m}}}^{2}}$$

where c_predicted, i_ = predicted plasma concentration, c_observed, i_ = observed plasma concentration, m = number of observed values.

## Results

The study sample consisted of 33 participants, 43% female, with a median (minimal and maximal range) age of 54.5 (20–85) years (Table I). All individuals had a history of traumatic brain injury and had undergone neurosurgical procedures. At the time of vancomycin administration, the median (minimal and maximal range) glomerular filtration rate was 105 (29.0–149.9) mL/min. Other clinical and biochemical information of this cohort was published by Tuon *et al*. [12].

**Fig. 2:**
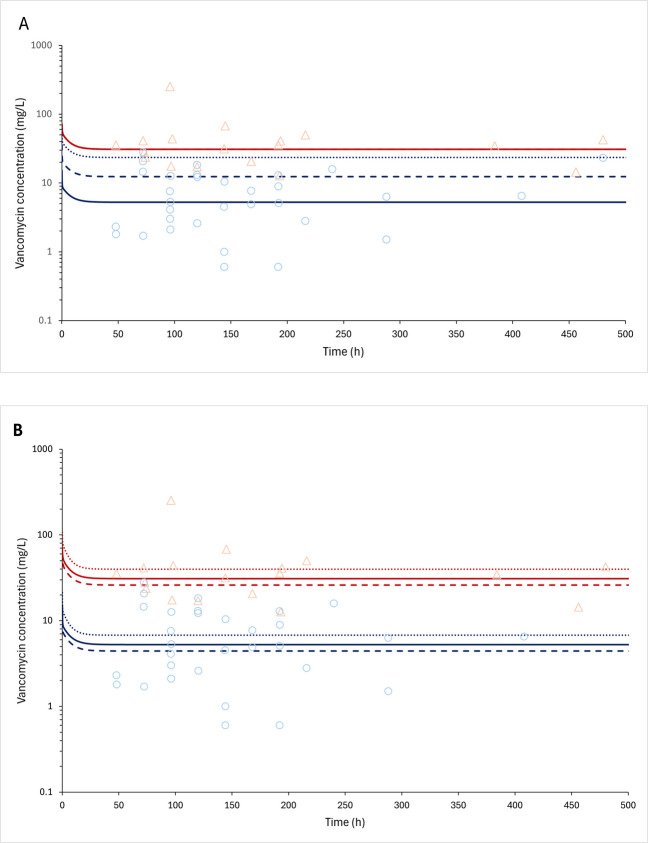
Vancomycin PBPK model fitting of the Therapeutic drug monitoring (TDM) study dataset of patients with ventriculitis (Tuon *et al*. [12]). Predicted and observed plasma and cerebrospinal fluid (CSF) vancomycin pharmacokinetic profiles after an intravenous loading dose of 30 mg/kg followed by 60 mg/kg intravenous continuous doses every 24 h. The simulated plasma and CSF concentration *versus* time profiles in one individual are shown as red and blue lines, respectively. The observed plasma and CSF concentrations from the TDM study are plotted as light red triangles and light blue dots, respectively. (**A**) Evaluation of plasma-to-interstitial brain partition coefficient (PC_CSF_) value: solid line: 0.17; dashed line: 0.40 and dotted line: 0.77, the plasma concentrations overlap, fraction unbound in plasma (fup) set as 0.67. (**B**) Evaluation of fup value: solid line: 0.67; dashed line: 0.80 and dotted line: 0.40; PC_CSF_ value set as 0.17.

In the base brain PBPK model, the distribution to the tissues was perfusion-limited with tissue-to-plasma coefficients predicted using the Rodgers and Rowland equations, with the PC_CSF_ value of 0.77 (dimensionless) calculated using Eq. 2. Nevertheless, this approach was not adequate to describe the vancomycin ventricular CSF concentrations in patients with ventriculitis (Fig. 2). To evaluate the uncertainties of input data on ventricular CSF vancomycin exposure, a sensitivity analysis was performed, evaluating the influence of 38 brain-related input parameters, fraction unbound to plasma protein and water volume in plasma (Eq. 2) on CSF Area Under the Curve (AUC_0-t;CSF_) and CSF maximum concentration (Cmax_CSF_) (Table III).

**Table III:** Results of Sensitivity Analysis of Brain-specific Input Parameters On Vancomycin Cerebrospinal Fluid (CSF) Exposure Considering the PBPK Model in Ventriculitis Patients

Input parameter^a^	AUC_0-t;CSF_	Cmax_CSF_
	Sensitivity, ranked by value	
Plasma-to-interstitial brain partition	0.99	0.63
Plasma-to-interstitial brain surface area	0.00	0.33
Small endothelial pores diameter	0.00	0.33
Brain volume	0.02	0.02
Albumin CSF to plasma ratio^b^	0.00	0.00
Fraction unbound in plasma protein^b^	−0.90	−0.65
Fraction of water in CSF^b^	0.00	0.00
Fraction of water in plasma^b^	0.0002	0.02

*AUC*_*0-t;CSF*_  CSF Area Under the Curve in the administration interval, *Cmax*_*CSF*_ CSF maximum concentration

^a^Other brain input parameters that had no or minimal influence on vancomycin exposure are not shown in this table

^b^Input parameters considered in plasma to interstitial brain partition equation

Based on the input parameters evaluated during the sensitivity analysis (Table III), the PC_CSF_ and fu were identified as the most relevant factors and were selected for further model refinement using the parameter identification tool. Figure 2A demonstrates the plasma and ventricular CSF concentration *versus* time profiles during PC_CSF_ optimization. Thus, the optimal PC_CSF_ value estimated was 0.17 (dimensionless) using the observed vancomycin ventricular CSF concentrations in patients with ventriculitis to fit this analysis. Figure 2B demonstrates the impact of fup on plasma and CSF concentration simulations. The fup value of 0.67 was set in the final model. A high interindividual variability in the vancomycin concentrations in plasma and CSF was observed in the patients with ventriculitis under vancomycin continuous infusion administration (Fig. 2).

**Fig. 3:**
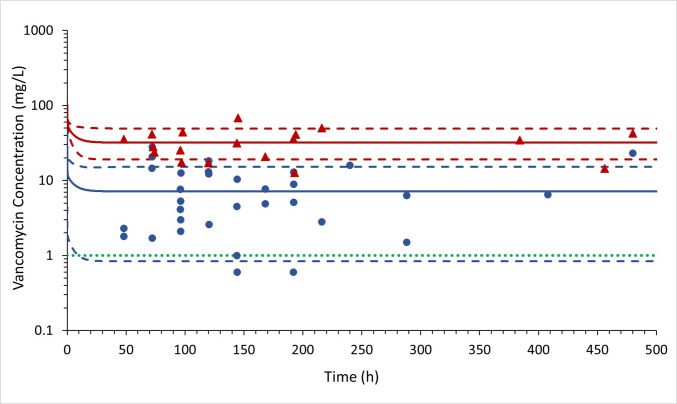
Predicted and observed (Tuon *et al*. [12]) plasma and cerebrospinal fluid (CSF) vancomycin pharmacokinetic profiles after an intravenous loading dose of 30 mg/kg followed by 60 mg/kg intravenous continuous doses every 24 h. The mean simulated plasma and CSF concentration *versus* time is shown as solid red and blue lines, respectively. The 5 and 95 percentiles for plasma and CSF simulated concentrations are demonstrated as dashed red and blue lines, respectively. The observed plasma and CSF concentrations from the therapeutic drug monitoring study (Tuon *et al*. [12]) are plotted as red triangles and blue dots, respectively. The green dashed line represents 1 mg/L vancomycin concentration.

Interindividual variability was then incorporated into the PBPK model by simulating 33 virtual individuals with 43% female, age range of 20 to 85 years old and glomerular filtration rates varying from 29 to 188 mL/min, matching the characteristics of patients from Tuon *et al*. [12]. Due to the high coefficient of variation of 85.5% calculated in the clinical steady-state ventricular CSF concentrations, an increased variability in PC_CSF_ parameter was incorporated as 0.17 ± 0.14 (Fig. 3). Mean simulated vancomycin concentrations in plasma and ventricular CSF in ventriculitis patients were 32.00 mg/L and 7.15 mg/L, respectively. The observed/predicted ratios for plasma and ventricular CSF concentrations were 1.1 and 0.8, respectively. The predicted ventricular CSF/plasma concentration ratio was 0.22, which closely matched the observed ratio of 0.17 (Table IV).

**Table IV:** Predicted and Observed Plasma and Cerebrospinal Fluid (CSF) Steady State Concentrations and CSF/plasma Ratios in Patients With Ventriculitis

Parameters (Unity)	Mean Values (minimal – maximal)
**Internal validation – TDM study with ventriculitis patients**^**a**^	
**Plasma**	
Css obs (mg/L)	35 (12.7–67.9)
Css pred (mg/L)	32 (18.1–66.6)
*Ratio Css obs/Css pred*	*1.1*
**MRD**	**1.9**
**Cerebrospinal fluid**	
Css obs (mg/L)	5.8 (0.6–27.9)
Css pred (mg/L)	7.2 (0.17–16.3)
*Ratio Css obs/Css pred*	*0.8*
**MRD**	**3.0**
CSF/plasma Ratio obs	0.17
CSF/plasma Ratio pred	0.22
*Ratio obs/pred*	*0.77*
**External validation – ventriculitis patients from Albanese** *et al. * [15]^**b**^	
**Plasma**	
Css obs (mg/L)	24 (11.6–46.8)
Css pred (mg/L)	28.4 (20.5–41.7)
*Ratio Css obs/Css pred*	0.8
**MRD**	**1.5**
**Cerebrospinal fluid**	
Css obs (mg/L)	3.3 (1.6–15.5)
Css pred (mg/L)	6.5 (1.1–12.0)
*Ratio Css obs/Css pred*	*0.5*
**MRD**	**2.1**
CSF/plasma Ratio obs	0.14
CSF/plasma Ratio pred	0.23
*Ratio obs/pred*	*0.61*

*CSF* cerebrospinal fluid, *Css* concentration at steady-state,* obs* observed data, *pred* predicted data, *TDM* therapeutic drug monitoring, *MRD* mean relative deviation

^a^intravenous loading dose of 30 mg/kg followed by 60 mg/kg intravenous continuous doses each 24 h

^b^intravenous loading dose of 15 mg/kg followed by 62 mg/kg intravenous continuous doses each 24 h

**Fig. 4:**
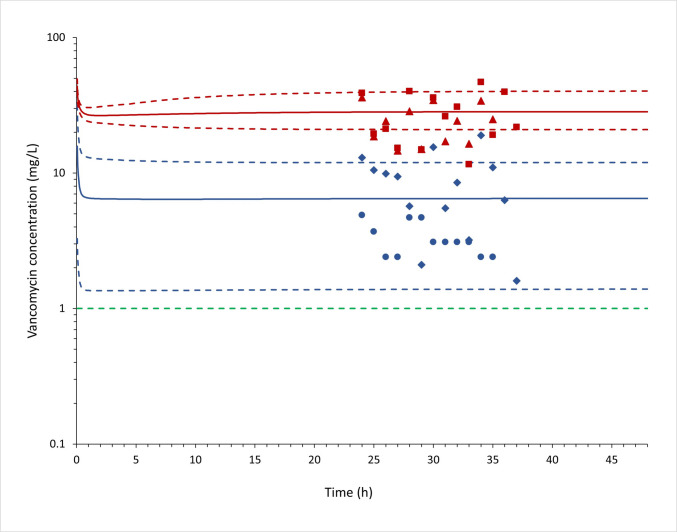
Predicted and observed (Albanese *et al*. [15]) plasma and cerebrospinal fluid (CSF) vancomycin pharmacokinetic profiles after an intravenous loading dose of 15 mg/kg followed by 62 mg/kg intravenous continuous doses every 24 h. The mean simulated plasma and CSF concentration *versus* time are shown as solid red and blue lines, respectively. The 5 and 95 percentiles for plasma and CSF simulated concentrations are demonstrated as dashed  red and blue lines, respectively. The observed plasma and CSF concentrations in ventriculitis patients (Albanese *et al*. [15]) are plotted as red triangles and blue dots, respectively. The observed plasma and CSF concentrations in meningitis patients (Albanese *et al*. [15]) are plotted as red squares and blue diamonds, respectively. Observation: The timing of observed concentrations was arbitrarily selected, as not reported by Albanese *et al*. [15], but assumed to reflect steady-state conditions. The green dashed line represents 1 mg/L vancomycin concentration.

To perform an external validation, the vancomycin PBPK brain model was applied to simulate a clinical study that stratified patients with ventriculitis or meningitis [15] (Fig. 4). Due to the lack of demographic and clinical information of the participants, the simulation of this study was performed with 13 virtual individuals with 50% female (assumed), with an age range of 25 to 58 years old. The observed data in plasma and ventricular CSF from ventriculitis patients [15] were compared with the simulation results and are demonstrated in Table IV.

The MRD values (Table IV) for the model in plasma were found to meet the acceptance criteria; however, for the CSF concentrations, the values were higher than 2 for both internal and external validation. Residuals *versus* time plots are demonstrated in Figures S1 (internal cohort) and S2 (external cohort; [15]), indicating the PBPK model performance. 

## Discussion

Vancomycin is a large, hydrophilic molecule commonly used to treat CNS infections caused by Gram-positive bacteria. However, the specific contributions of plasma-to-interstitial brain partitioning, plasma protein binding, and brain-related physiological factors to its penetration into the CNS have not been systematically evaluated. This study mechanistically demonstrated the vancomycin penetration into lateral ventricular cerebrospinal fluid in patients with ventriculitis using a PBPK approach.

The entry of drugs into the CNS is governed by molecular size, lipophilicity, plasma-protein binding, and active transport [17]. Brain parenchyma, interstitial fluid (ISF), and the multiple CSF compartments are in continuous—though asymmetric—exchange of water and solutes, which further complicates the prediction of local drug concentrations. Vancomycin, the drug examined here, is highly hydrophilic (logP =  − 3.10), possesses a large molecular mass (1,449 Da), and has a fup of roughly 0.67. Into the PBPK model, the large molecule module better described the vancomycin penetration into the brain when compared to the small molecule module, which is based on passive diffusion distribution.

As a simplification of the model, we assumed that vancomycin concentrations in CSF are the same in the ISF compartment, but the differences between these compartments should be considered [18]. Once vancomycin reaches the CNS, bulk-flow dynamics dominate: CSF turns over more rapidly than ISF and effectively “pulls” solutes from the parenchyma [18]. Because CSF is cleared to the systemic circulation faster than solutes move from ISF to CSF, a sink effect arises in which ventricular CSF concentrations are typically lower than those in the surrounding ISF [18]. In this study, all CSF samples were obtained via an EVD; sampling directly from the ventricles may attenuate but does not abolish the difference between ventricular CSF and ISF vancomycin concentrations. Lastly, to add complexity, the role of brain drug transporters on vancomycin disposition is not reported.

The pathophysiological differences between meningitis and ventriculitis significantly influence drug penetration across the BBB, affecting therapeutic efficacy. In bacterial meningitis, intense inflammation disrupts the BBB by opening tight junctions between endothelial cells, increasing permeability and facilitating the entry of hydrophilic and large-molecule antibiotics into CSF [17]. The reported vancomycin plasma to CSF penetration in meningitis varied from 0.29 to 0.80 [15, 19–21].

Conversely, ventriculitis, typically linked to indwelling ventricular devices, generally produces less intense meningeal inflammation than meningitis, resulting in reduced passive diffusion of antibiotics into the CSF. Moreover, when an EVD is inserted after head trauma or neurosurgery, the BBB may be disrupted, whereas patients whose only intervention is EVD placement may retain a comparatively intact BBB. These differing clinical contexts likely underlie the wide range of reported vancomycin plasma-to-CSF penetration ratios in ventriculitis, which span 0.03–0.70, with mean values clustering around 0.15–0.17 [12, 15, 22–24].

In sensitivity analysis performed during vancomycin PBPK brain model refinement, we mechanistically showed the sensitivity of changes in the parameters considered in Eq. 2, albumin CSF to plasma ratio, fraction unbound in plasma, fraction of water in CSF and fraction of water in plasma, on PC_CSF_ value. The vancomycin fup is highly variable among critically ill patients, varying from 0.47 to 0.92 and a mean of 0.67 [25]. The changes in fup value impact vancomycin plasma and CSF levels (Fig. 2B). To address all possible sources of variability in vancomycin CSF penetration, the PC_CSF_ value was adjusted using the identification parameter tool (Fig. 2). Besides the changes in fup, a plausible hypothesis is that efflux transporters may play a role in the markedly low penetration of vancomycin into the CSF in patients with ventriculitis. In this case, the input of an efflux clearance from the brain to plasma mediated by drug transporters into the PBPK model could improve the predictions. There are a few studies demonstrating the role of drug transporters in vancomycin disposition in the kidney [26, 27], but not in the brain. Future work should address the role of brain drug transporters in vancomycin disposition.

There are other vancomycin PBPK models published; some considerations should be taken to distinguish them from the present model. Previously published PBPK models primarily addressed vancomycin disposition in populations other than patients with ventriculitis. For example, Emoto *et al*. [28] evaluated specific subgroups such as Japanese adults, paediatric patients, and individuals with renal or hepatic impairment; Shuai *et al*. [29] and Cao *et al*. [30] assessed critically ill neonates; and Radke *et al*. [31] and Olivo *et al*. [32] focused on septic patients. Our model is grounded in Rezende *et al*. [13], developed by our research group, which focused on vancomycin-induced nephrotoxicity and the derivation of toxicological parameters.

To accurately characterise vancomycin tissue penetration, the present model employed the large-molecule module, since the small-molecule module was insufficient to represent the distribution of a high-molecular-weight compound such as vancomycin. This strategy allowed preservation of the drug’s physicochemical property of logP = –3.1, consistent with its highly hydrophilic behaviour.

Among previous studies, only Emoto *et al*. [28], using Simcyp v.16, retained this experimentally supported logP value. In most other models, the fitting of vancomycin predicted profile with observed data was achieved by markedly increasing the logP value, with reported estimates ranging from 2.26 to 23.1 [28–33] while applying the small-molecule module. The partition coefficient from plasma to tissue value was optimised in some models, such as Rezende *et al*. [13] for kidney penetration, the present model for CSF penetration and Olivo *et al*. [32] for kidney, liver, lung and subcutis penetration. Regarding protein binding, the current model adopted a fup value of 0.67, in line with Radke *et al*. [31], Cao *et al*. [30], Shuai *et al*. [29], and Olivo *et al*. [32]. In contrast, Emoto *et al*. [28] and Maruyama *et al*. [33] implemented a lower fup value of 0.45.

Summarising, the present PBPK model maintained the LogP and hydrophilic characteristics of the molecule, using a large molecule model that can better capture vancomycin disposition and focus on describing permeability to CSF.

After model refinement, the simulations (Fig. 3) with the vancomycin PBPK model predicted the observed ventricular CSF and plasma concentration *versus* time course in patients with ventriculitis [12] reasonably well (Table IV), considering the high interindividual variability.

The AUC_0–24 h_ divided by the minimal inhibitory concentration (MIC) of 400 to 600 (assuming a vancomycin MIC of 1 mg/L) is currently considered the optimal pharmacodynamic (PD) target to achieve clinical efficacy and microbiological cure if systemic *Staphylococcus aureus* is suspected/proved [3]. Nevertheless, there is less clarity about the relevant drug exposure at the target site of infection (e.g. the ventricular CSF). Vancomycin is recommended 30–60 mg/kg/day for meningitis and ventriculitis to ensure sufficient CSF concentrations [34]. Despite the high interindividual observed and predicted vancomycin steady-state CSF concentrations (Fig. 3), the intravenous loading dose of 30 mg/kg followed by 60 mg/kg intravenous continuous doses every 24 h is a reasonable dosing proposal when alternative options are not available. Nevertheless, more work is required to understand the PK/PD target at the site of infection for patients with ventriculitis. It’s important to note that patients with vancomycin plasma AUC_0–24 h_ > 600 mg*h/L increase the risk of renal injury [3]. If the site-specific PD target corresponded only to the MIC target, there would be little justification for raising vancomycin exposure to 60 mg/kg/day, thereby avoiding an unnecessary increase in nephrotoxicity risk. By contrast, the Infectious Diseases Society of America ventriculitis guidelines recommend achieving CSF concentrations 10–20 times the MIC when intraventricular therapy is selected [34]. Therefore, there is an urgent need to establish a PD target in patients with ventriculitis.

This study has several limitations. First, the sample size was relatively small. However, it is important to note that cerebrospinal fluid vancomycin measurements in patients with ventriculitis represent a rare and clinically uncommon dataset. Despite this limitation, the model demonstrated strong predictive performance, accurately characterizing vancomycin kinetic disposition within this unique patient population. Second, the fup, a key determinant of vancomycin disposition, was not directly measured in this cohort. Nonetheless, we incorporated a fup value that is widely used in PBPK models, thereby providing a reasonable and evidence-supported approximation for the analyses. Third, potential drug–drug interactions with concomitant medications were not evaluated. In addition, other biochemical and clinical characteristics that may influence vancomycin disposition were not incorporated into the analysis. Finally, the PBPK model was not able to fully capture the interindividual variability in vancomycin penetration into brain. *In vitro* studies addressing the transport of vancomycin through BBB could help in this issue.

Although the present PBPK model shows good potential for optimizing the treatment of ventriculitis, the lack of robust clinical data correlating model parameters with therapeutic outcomes limits its immediate application. Future research should focus on prospective studies with a significant number of rigorously monitored patients in order to establish the foundation for the PK/PD vancomycin target for safe and effective implementation of dosing optimization in the treatment of central nervous system infections using PBPK modeling and simulation.

## Conclusion

The low and variable penetration of vancomycin into the CNS highlights the critical role of therapeutic drug monitoring and individualized therapy in the management of patients with ventriculitis. This study demonstrated that the integration of the physicochemical and ADME properties of vancomycin with human physiological data in a PBPK model was able to predict vancomycin penetration into the ventricular CSF, offering a promising platform for dose adjustment in patients with ventriculitis. The results of this study represent a significant advancement in the understanding of vancomycin pharmacokinetics in patients with ventriculitis and may serve as a basis for the development of therapeutic strategies.

## Supplementary Information

Below is the link to the electronic supplementary material.

ESM 1 (PDF 53.6 KB)

## Data Availability

The data sets obtained and/or analyzed during the current study are available from the corresponding author upon reasonable request.
