# Supplementary material for: Assessment of Vancomycin Penetration into Cerebrospinal Fluid in Patients with Ventriculitis Using a Physiologically Based Pharmacokinetic Approach
Source: Pharm Res. 2026 Feb 4;43(2):345–55. doi: 10.1007/s11095-026-04023-5 (PMC12963244; doi:10.1007/s11095-026-04023-5)
Supplement: Supplementary file 1 — (PDF 53.6 KB) [file 11095_2026_4023_MOESM1_ESM.docx]

**Supplementary Material**

Figure S1: Residuals (Simulation value minus Observed value) versus time plot of predicted and observed (Tuon et al. [1]) plasma and cerebrospinal fluid (CSF) vancomycin pharmacokinetic profiles after an intravenous loading dose of 30 mg/kg followed by 60 mg/kg intravenous continuous doses every 24h. The plasma and CSF residuals are plotted as red triangles and blue dots, respectively.


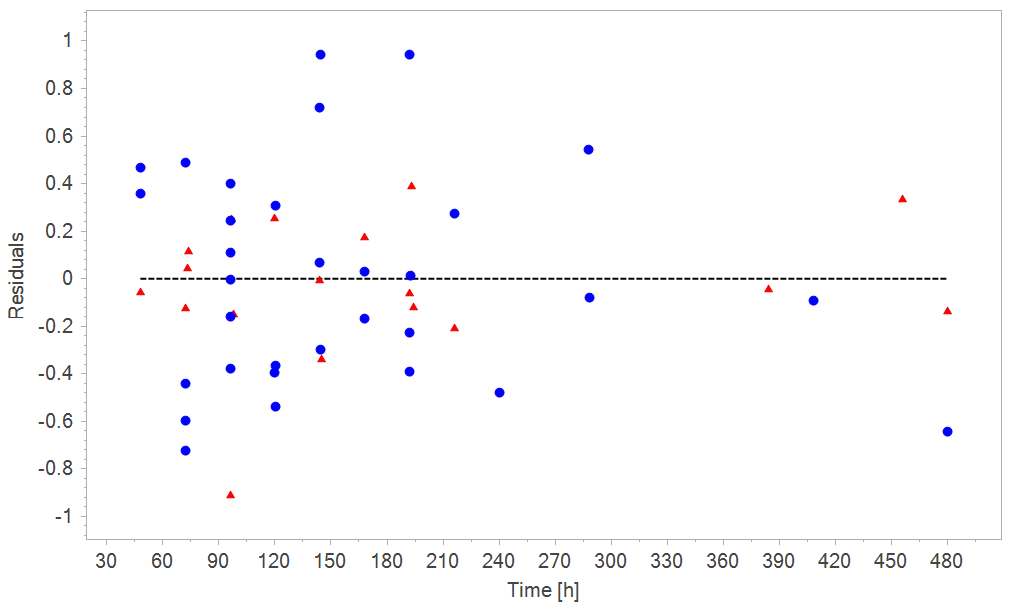


Figure S2: Residuals (Simulation value minus Observed value) versus time plot of predicted and observed (Albanese et al. [2]) plasma and cerebrospinal fluid (CSF) vancomycin pharmacokinetic profiles after an intravenous loading dose of 15 mg/kg followed by 62 mg/kg intravenous continuous doses every 24h. The plasma and CSF residuals are plotted as red triangles and blue dots, respectively.


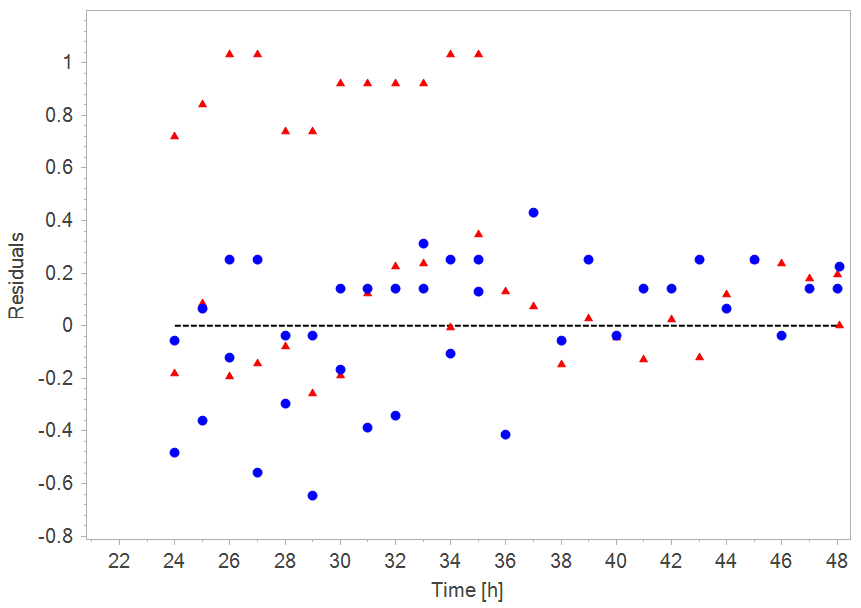


References

1. Tuon FF, Yamada CH, Cieslinski J, Dos Santos Oliveira D, Ribeiro VST, Gasparetto J, et al. Cerebrospinal Fluid Penetration of Vancomycin During Continuous Infusion Therapy in Patients With Nosocomial Ventriculitis. Ther Drug Monit. 2021;43(6):807-11. <https://doi.org/10.1097/FTD.0000000000000907>.
2. Albanèse J, Léone M, Bruguerolle B, Ayem ML, Lacarelle B, Martin C. Cerebrospinal fluid penetration and pharmacokinetics of vancomycin administered by continuous infusion to mechanically ventilated patients in an intensive care unit. Antimicrob Agents Chemother. 2000;44(5):1356-8. <https://doi.org/10.1128/AAC.44.5.1356-1358.2000>.
